# Detritus and larval competition influence phenotypic traits, nutrient stoichiometry, and vector competence for dengue virus in *Aedes aegypti*

**DOI:** 10.1186/s13071-026-07467-1

**Published:** 2026-06-02

**Authors:** Limarie J. Reyes-Torres, Donald A. Yee, Barry W. Alto

**Affiliations:** 1 School of Biological, Environmental, and Earth Sciences, The University of Southern Mississippi, Hattiesburg, MS USA; 2 Florida Medical Entomology Laboratory, Institute of Food and Agricultural Sciences, University of Florida, Vero Beach, FL USA

**Keywords:** *Aedes aegypti*, *Aedes mediovittatus*, Dengue virus, Vector competence, Larval competition, Detritus inputs, Nutrients, Stable isotopes, Phenotypic traits

## Abstract

**Background:**

*Aedes aegypti* is the primary vector of dengue virus (DENV), a major arboviral disease affecting human health. Variation in detritus inputs and larval competition in containers where the species develop can influence female *Ae. aegypti* phenotypic traits, nutrient content (carbon, nitrogen, C:N ratio), and elemental isotopes (δ^13^C and δ^15^N), ultimately affecting vector competence for DENV.

**Methods:**

Female *Ae. aegypti* were reared under simulated urban detritus levels (low, medium, and high) and larval intra- (30:0 and 60:0) and interspecific (30:30 and 60:60) competition with *Ae. mediovittatus* (*Ae. aegypti*: *Ae. mediovittatus*). *Aedes aegypti* were offered a DENV-1–infected bloodmeal. Transmission assays were conducted, followed by assessment of susceptibility to infection, disseminated infection, and transmission rates. Body nutrient composition and isotope values were measured and analyzed in relation to phenotypic traits and vector competence for DENV-1, with male body nutrient profiles used as proxies for females where sample sizes were limited.

**Results:**

Longer development time and reduced wing length in *Ae. aegypti* were associated with low detritus. Wing length increased under interspecific competition with *Ae. mediovittatus* but decreased under intraspecific competition. Higher δ^15^N values in *Ae. aegypti* body tissues were associated with greater detritus availability and with interspecific competition. DENV-1 dissemination exhibited a nonlinear relationship with δ^15^N, showing high dissemination at low δ^15^N, the lowest dissemination at intermediate δ^15^N, and an increase at higher δ^15^N. Vector competence for DENV-1 was reduced under conditions of low detritus and low intraspecific competition (30:0). High interspecific competition combined with high detritus negatively affected *Ae. aegypti* phenotypic traits and reduced DENV-1 vector competence. Under the most extreme conditions (high interspecific competition, 60:60, and high detritus), *Ae. aegypti* failed to reach adulthood. At intermediate detritus levels for the same density, *Ae. aegypti* exhibited high rates of DENV-1 disseminated infection.

**Conclusions:**

This research underscores the importance of larval crowding and nutritional availability experienced during the immature stages of *Ae. aegypti* on adult nutrient stoichiometry and DENV-1 infection. These findings suggest the potential use of δ^15^N as a tool to assess how larval nutritional and competitive environments shape dengue vector competence, with implications for understanding and predicting *Ae. aegypti*-driven DENV-1 dynamics in urban and suburban areas. Interpretations should consider that female nutrient estimates were partially inferred from male data due to low female sample sizes, survivorship was estimated assuming a 1:1 sex ratio, and some high-mortality treatments lacked surviving adults.

**Graphical Abstract:**

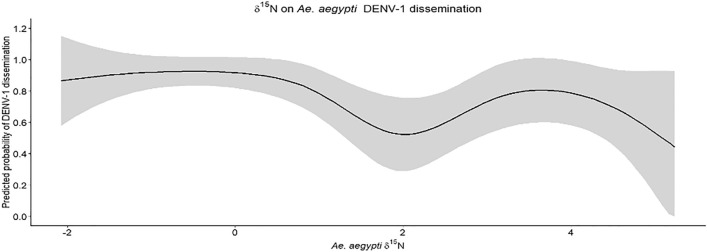

**Supplementary Information:**

The online version contains supplementary material available at 10.1186/s13071-026-07467-1.

## Background

Understanding the environmental determinants of pathogen transmission is a critical challenge for both research and public health, particularly given the recent emergence and reemergence of infectious diseases [1, 2]. Environmental factors can influence arthropod vectors by shaping their abundance, phenotypes, fitness, and vector competency for pathogens. Vector competence (VC) is defined as the capacity of acquiring and transmitting a pathogen [3, 4], and for arthropods, depends on susceptibility to infection, disseminated infection, and transmission, usually by bite. Dengue virus (DENV) is one of the most important arthropod-linked diseases concerning human health [5–7]. The virus has four serotypes (DENV 1–4), and its mosquito vectors have a wide geographical distribution and continue to spread effectively and rapidly due to urbanization, globalization, lack of effective mosquito control measures, and climate change [5–7]. *Aedes aegypti* (yellow fever mosquito) females are the primary vector of the virus and transmit it to humans during probing and blood feeding, which they require for egg maturation [7–9].

Vector research primarily focuses on the environmental conditions experienced by the adult stage of the vector. However, the conditions experienced by immature stages may be equally, or in some cases, more important in determining vector-borne disease transmission as larval competition effects can be carried over to the mosquito adult stage and reflect lower vector fitness and changes in VC [4, 10–12]. Increased larval density has been associated with reduced adult *Ae. aegypti* body size [13], reduced survival to adulthood [13], and prolonged development times under intra- [14, 15] and interspecific [15] competition. *Aedes aegypti* larvae that experienced high intraspecific competition were likely to exhibit higher Sindbis virus disseminated infection than individuals from low competition conditions [4]. A similar pattern was observed when *Ae. aegypti* from low density environments showed lower susceptibility to infection and disseminated infection with DENV-2 compared with females from higher inter- and intraspecific larval competition with the Asian tiger mosquito *Ae. albopictus* (*Ae. albopictus*: *Ae. aegypti*) [10].

In aquatic containers where mosquitoes develop, allochthonous detrital inputs from plant and animal sources vary in their carbon (C) and nitrogen (N) content and form the base of mosquito-dominated food webs [16]. Nitrogen is often the limiting resource in larval habitats, and nitrogen-rich diets, such as animal detritus, generally enhance *Aedes* spp. phenotypic traits, including shorter development time, greater biomass or wing length, and higher survival [13, 16, 17]. Beyond shaping mosquito life-history traits, nutrient availability in container habitats can also influence physiological processes associated with arbovirus VC [12, 13, 18, 19]. However, relatively few studies have explicitly evaluated the relationship between environmentally derived nutrients and VC in *Ae. aegypti*. Increased inputs of leaf and animal detritus have been associated with higher larval body δ^15^N and biomass [18], elevated adult body percent nitrogen (%N) [12, 19], larger adult body size [13], faster development time [12, 13], greater survival to adulthood [12, 13], increased capacity for vertebrate blood feeding [13], and reduced Zika virus dissemination [12, 19] and transmission in *Ae. aegypti* [12]. In contrast, larval starvation or nutritional stress prolongs development time [13], produces smaller adults [20, 21], reduces fecundity [13, 21], and compromises midgut barrier integrity through thinning of the basal lamina [20]. These nutritionally constrained mosquitoes can also exhibit increased Zika virus midgut escape and dissemination [20], as well as suppressed immune responses [22], e.g., to dengue virus infection [21]. Taken together, these findings indicate that variation in larval nutrition can have cascading effects on adult mosquito physiology and phenotypes and ultimately influence arbovirus VC.

Allochthonous detritus inputs [18], as well as detritus-mediated habitat conditions in larval containers [18, 23, 24], can vary across urban landscapes, reflecting economic and cultural activities that shape land-use patterns and local environmental heterogeneity [5, 25, 26]. Fine-scale environmental variation around containers—such as the extent of house abandonment, impervious surface cover, and canopy cover—has been shown to affect detritus type and biomass, as well as associated nutrient inputs (carbon, nitrogen, δ^13^C, and δ^15^N) across urban gradients [18, 27]. These differences in detrital resources in container habitats subsequently influence *Ae. aegypti* larval phenotypic traits and body nutrient composition [18]. Building on this premise, we evaluated how larval environmental conditions shape phenotypic traits and vector competence (VC) from a nutritional perspective by integrating resource availability and larval competition within an urban landscape context, where *Ae. aegypti* commonly occurs. Using an experimental approach grounded in ecological stoichiometry (body carbon [%C] and nitrogen [%N], and C:N ratios) and stable isotope analyses (δ^13^C and δ^15^N), we aimed to link *Ae. aegypti* larval nutrient assimilation across detritus treatments representative of an urban gradient (negative relationship between total detritus and indicators of urbanization) [18] and varying competition regimes to adult VC for DENV-1.

We hypothesized that low detrital inputs would lead to a decrease in *Ae. aegypti* female wing length (i.e., an indicator of net growth), lengthen development time, and have little influence on survival when compared with medium and high detrital inputs. We also hypothesized that low detrital inputs would decrease *Ae. aegypti* percent carbon (%C and δ^13^C) and nitrogen content (%N and δ^15^N) when compared with medium and high detrital inputs. We predicted that *Ae. aegypti* in interspecific competition with *Ae. mediovittatus* [18, 28], a stronger larval competitor [15], would exhibit reduced survival, shorter wing length, and prolonged development time, with these effects becoming more pronounced under low and medium detritus conditions. We also hypothesized that nitrogen content (%N and δ^15^N) of *Ae. aegypti* would decrease with higher *Ae. mediovittatus* density, with stronger effects in low and medium detritus treatments where we expected nitrogen amounts (limiting resource) to be lower. Lastly, we hypothesized that the combination of less detritus and the occurrence of interspecific competition with *Ae. mediovittatus* would decrease the nutrient profile and increase the VC of *Ae. aegypti* for DENV-1. Specifically, we predicted that *Ae. aegypti* females from the low detritus treatment level with interspecific competition would have lower %C and %N content in their body, lower δ^13^C and δ^15^N, and higher VC for DENV-1 than females from other treatment levels.

## Methods

### Larval competition experiments

Experimental containers were housed inside a climate-controlled room (photoperiod 12:12 Light:Dark, temperature 27 °C, 80% Relative Humidity) located at the Florida Medical Entomology Laboratory, University of Florida (UF-FMEL) in Vero Beach, Florida. Rectangular (35.5 cm L × 27.94 cm W × 8.26 cm H) plastic containers were filled with 1.86 L of deionized (DI) water and 5 mL of filtered tire water collected from the field as an inoculum containing microorganisms. Low, medium, and high urban density environments were simulated by using different detritus treatment levels including animal and plant sources (Table 1) found in containers across an urban gradient in Puerto Rico, USA [18]. Senescent plant detritus was dried in an oven for at least 48 h at 50 °C and weighed according to each treatment level (Table 1). Total detritus amount and detritus diversity per container were selected to form a gradient negatively associated with urban density (Table 1) on the basis of previously reported negative relationships between total detritus, vegetation *α* diversity, and indicators of urbanization such as impervious surface [18]. Additionally, studies have extensively documented that vegetation diversity, including plant species richness, generally declines with increasing urbanization [25, 29]. *Gryllodes sigillatus* (cricket) and *Drosophila melanogaster* (flies) were used as animal detritus sources [30, 31]. Freeze-dried *G. sigillatus* (Fluker Farms, Port Allen, LA) and live flies (Burlington, NC) were commercially acquired. Flies were reared following the Carolina *Drosophila* care guide [32] and cold-killed before drying.

**Table 1:** Detritus and larval competition treatments

Urban level	Detritus level	Detritus combinations	Total detritus (g)	AE:AM density
High	Low	G:D:M (0.150 g each)	0.45	30:0; 60:0; 30:30; 60:60
Medium	Medium	G:D:A:M (0.225 g each)	0.90	30:0; 60:0; 30:30; 60:60
Low	High	G:D:A:F:M (0.360 g each)	1.80	30:0; 60:0; 30:30; 60:60

*AE*  *Ae. aegypti*, *AM*  *Ae. mediovittatus*, *G* *Gryllodes sigillatus* (cricket), *D* *Drosophila melanogaster* (flies), *A* *Albizia lebbeck* (Siris), *F* *Ficus virens* (white fig), *M* *Mangifera indica* (mango). A 1:1 sex ratio was assumed for all replicates; therefore, estimates of female survival are based on this assumption and should be interpreted with this limitation in mind

Different combinations of *Ae. aegypti* and *Ae. mediovittatus* larvae were used to assess the influence of intra- and interspecific competition on *Ae. aegypti* (Table 1). Experimental larval densities were selected on the basis of data of a previously published field sampling of artificial containers in San Juan, Puerto Rico [18]. Field-observed larval densities of *Ae. aegypti* and *Aedes mediovittatus* were highly right-skewed, indicating that most sampled containers supported relatively low larval densities while a small number exhibited extremely high densities. Median densities were 68.5 and 30 larvae/L, and 75th percentiles (third quartile) were 192.8 and 119 larvae/L for *Ae. aegypti* and *Ae. mediovittatus*, respectively. The established laboratory larval densities fell below the upper quartile of field-observed densities and represent ecologically realistic conditions. Mixed-species treatments were initiated at equal densities to facilitate interpretation of interspecific competitive effects independent of initial abundance. *Aedes mediovittatus* was selected as a larval competitor due to its co-occurrence with *Ae. aegypti*, including sharing the same larval habitat, and its competence as a vector of DENV [18, 28, 33, 34] In Puerto Rico, *Ae. aegypti* is commonly found across the urban gradient whereas the endemic *Ae. mediovittatus* (Caribbean tree hole mosquito) inhabits less disturbed areas such as forests and low-density housing within the same urban gradient [18, 28, 33, 34]. The co-occurrence of both *Aedes* species is important, as it may lead to larval competition in shared larval container habitats and differences in VC for DENV-1.

Colonies of *Ae. aegypti* and *Ae. mediovittatus* were established at the UF-FMEL from eggs provided by the Centers for Disease Control and Prevention in San Juan, Puerto Rico. Eggs originated in Puerto Rico and were of mixed generations (generation number unknown) from colony-reared species. Although these colonies are maintained in the CDC laboratory, they are regularly supplemented with wild-caught individuals every 3–6 months (personal communication). While not a true field colony, the genetic background is not limited to the founding individuals associated with a long-established lab strain. An equal mixture of lactalbumin and *Saccharomyces cerevisiae* yeast were used to induce *Ae*. *aegypti* hatching [10, 35], and larvae were rinsed with deionized water before transfer to containers. Preliminary trials with *Ae*. *mediovittatus* showed high mortality when exposed to the lactalbumin and yeast mixture; perhaps indicating that the species has a higher sensitivity to water quality, e.g., total dissolved solutes. Therefore, *Ae*. *mediovittatus* eggs were hatched by being conditioned (egg papers kept damp) for 24 h and then submerged in clean water to induce hatching, which resolved issues with mortality. Four larval density treatment levels were established representing two levels of *Ae. aegypti* intraspecific competition and two levels of interspecific competition with *Ae. mediovittatus* (Table 1). A minimum of three replicates were conducted for each larval density by detritus treatment combination (Table 2). Treatments with higher mortality (based on preliminary experiments) included additional replicates to ensure sufficient mosquitoes for subsequent testing. Despite the unbalanced design (Table 2), statistical models that accounted for unequal variance and replication were used to ensure robust inference, e.g., multivariate analysis of variance (MANOVA) and analysis of variance (ANOVA). In addition, logistic regression does not require balanced sample sizes across groups; each observation contributes independently to the estimation of treatment effects, making the approach robust to differential replication among experimental conditions (see statistical analyses).

**Table 2:** *Aedes aegypti*:*Ae. mediovittatus* density and detritus treatment replicates

AE:AM density	Detritus	Replicates
30:0	L, M, H	7, 4, 5
60:0	L, M, H	5, 3, 3
30:30	L, M, H	4, 4, 3
60:60	L, M, H	3, 3, 3

*AE* *Ae. aegypti*, *AM* *Ae. mediovittatus*. Detritus treatments are *L* low, *M* medium, *H* high

*Aedes* sp. Pupae and adults were housed under the same environmental conditions as the larvae; containers were checked daily for pupae. Five to seven pupae were placed in 50 mL plastic tubes with deionized water and a cotton plug. Emergence date and adult sex were recorded, and adults were transferred to a cup cage (9 cm D × 14 cm H) with a mesh lid. Adults were cold anesthetized and sorted by species. *Aedes aegypti* males and females were placed in cup cages (9 cm D × 14 cm H) by treatment level and were provided a 10% sucrose solution via soaked cotton. The experiment was monitored until all mosquitoes emerged as adults or died in the immature stages. Survivorship to adult emergence was calculated for each treatment replicate by dividing the total number of adults by half the number of larvae of that species originally allocated to the container (survivorship proportion assumes a 1:1 sex ratio).

### Cell line propagation and DENV-1 inoculation

All virology methods were performed at the UF-FMEL biosafety level (BSL) 2 facility and followed standard operating procedures. Vero cells (African green monkey kidney cells) were propagated in tissue culture flasks (175 cm^2^) with media (Minimum Essential Medium supplemented with 10% fetal bovine serum, 2% penicillin–streptomycin, and 2% antibiotic–antimycotic solutions) in an incubator at 37 °C and a 5% CO_2_ atmosphere. A plaque assay using tenfold serial dilutions of viral stock was performed with Vero cells and DENV-1 (GenBank accession number JQ675358). The virus isolate originated from an infected human in 2010 in Key West, FL, USA and was passaged three times in Vero cells. Dengue virus serotype 1 (DENV‑1) was used in this study because it is well characterized in our laboratory, allowing for standardized infection protocols and reproducible results. DENV‑1 is also epidemiologically relevant, as it is commonly circulating and contributes substantially to the global dengue disease burden [5–7]. Additionally, the DENV-1 strain used here is comparable to strains employed in previous studies on *Aedes* mosquitoes [e.g., 36, 37], facilitating comparisons across studies.

Plaque assays [38] were used to determine the amount of DENV-1 stock to be used to infect the Vero cell cultures for the experiment. Tissue culture flasks (175 cm^2^) were seeded with 21.6 × 10^6^ Vero cells and media and maintained at 37 °C with 5% CO_2_. Subsequently, confluent monolayers of Vero cells were inoculated with DENV-1 at a multiplicity of infection (number of viruses per cell) of 0.01 plaque-forming units (PFU) per cell. The inoculated flask was rocked gently every 10 min for 1 h at 37 °C; 25 mL of media were added, and the flasks were then stored at 37 °C for 6 days before being used for the mosquito infection trials.

### Infection and transmission assays

A DENV-1 infectious bloodmeal with a titer of 5 × 10^6^ PFU/mL was prepared by combining 25 mL of defibrinated bovine blood with 25 mL of cell culture homogenate (1:1) 6 days postinfection (dpi) with DENV-1. The viral titer of infected blood was determined with blood samples taken before and after blood feeding. The bloodmeal titer of 5 × 10^6^ PFU/mL was slightly higher than typical human viremia levels (3.2 × 10^6^ PFU/mL; [39–41]), and within a biologically relevant range for mosquito infection experiments. Higher viral titers generally increase the likelihood of mosquito infection and subsequent dissemination, while lower titers may reduce infection rates [42, 43]. Therefore, the titer used represents an intermediate, biologically relevant dose that allows for meaningful assessment of DENV-1 vector competence in *Ae. aegypti*. Adenosine triphosphate (ATP) at a 0.005 M concentration was added as a phagostimulant during feeding trials. Using 6 dpi cells was determined to be effective on the basis of results presented in a previous study [44], where the DENV-1 isolate used in the current study showed high titers following propagation in Vero cells. Thus 7–10-day-old *Ae. aegypti* females were bloodfed for 1 h with a Hemotek (Lancashire, United Kingdom) membrane feeding system warmed to 37 °C. Parafilm-covered Hemotek discs were loaded with 3 mL of DENV-1 infected blood. Fully engorged females were placed in cup cages (9 cm D × 14 cm H) with meshed lids in an incubator under a 28 °C, 80% humidity, 12:12 L:D cycle for a 14-day incubation period to allow for virus replication and dissemination to secondary tissues and organs. A 10% sucrose solution was offered via soaked cotton pledgets.

Transmission assays consisted of collecting mosquito saliva in hematocrit capillary tubes 14 days after ingestion of the infected blood [45]. Briefly, *Ae. aegypti* females were cold anesthetized at 4 °C. Wings and legs were dissected within a glovebox using sterile forceps and the body of each female was then fastened to tape on a platform of acrylic plastic. The proboscis was inserted into a capillary tube containing mineral oil and each female was allowed to expectorate saliva for 1 h. Subsequently, the mineral oil and saliva were dispensed into 2 mL centrifuge tubes with 300 µL of Minimum Essential Media.

*Aedes aegypti* females were dissected and the abdomen was stored for viral testing while the head and thorax were stored for nutrient analyses. Although this approach was necessary for determining viral infection status, we acknowledge that it does not capture abdominal energetic reserves associated with the fat body. *Aedes aegypti* legs and saliva samples were stored separately in 2 mL centrifuge tubes at −80 °C for later DENV-1 testing and determination of susceptibility to infection, disseminated infection, and transmission rates, respectively; 1 mL of Minimum Essential Media was added to centrifuge tubes with the abdomens and legs and stored at −80 °C. Wings were stored in empty 2 mL centrifuge tubes at −80 °C for later wing length measurements.

### RNA isolation and PCR

Frozen *Ae. aegypti* abdomen and leg samples were thawed and lysed by the addition of two steel BB pellets to each centrifuge tube followed by homogenization using a TissueLyser II sample disruptor (Qiagen, Germantown, MD, USA) at 19.5 Hz for 3 min. Samples were then centrifuged at 13,200 rpm for 5 min at room temperature (15–25 °C). DENV-1 RNA from abdomens, legs, and saliva were extracted from a 140 μL aliquot of homogenate using the QIAamp Viral RNA Mini Kit (Qiagen, Valencia, CA) following the manufacturer’s protocol. The Bio-Rad CFX96 Touch Real-Time Polymerase Chain Reaction (PCR) Detection System was used to amplify and quantify the DENV-1 RNA along with DENV-1 specific primers and fluorogenic probe hydrolysis technology (TaqMan^®^, Applied Biosystems, Foster City, CA) [46]. Primers were designed to target the NS5 gene with the following sequences (5′˗ 3′): forward primer, GAC ACC ACA CCC TTT GGA CAA (genomic region 8586–8606) reverse primer, CAC CTG GCT GTC ACC TCC AT (genomic region 8692–8673), and probe, AGA GGG TGT TTA AAG AGA AAG TTG ACA CGC G (genomic region 8606–8638) (Integrated DNA Technologies, Coralville, IA). The program for qRT-PCR was 30 min at 60 °C and 2 min at 95 °C, followed by 39 cycles of 15 s at 95 °C and 60 s at 60 °C. Quantitative reverse transcription polymerase chain reaction (qRT-PCR) was performed using the SuperScript III Platinum One-Step qRT-PCR Kit (Invitrogen Life Technologies, Carlsbad, CA) with *Taq* mix, 2 × ThermoScript reaction mix, forward primer (10 μmol/L), reverse primer (10 μmol/L), fluorogenic probe (10 μmol/L), diethyl pyrocarbonate (DEPC)-treated water, and the sample extract. The reagent quantities for PCR used in testing mosquito samples are described in a previous study [35]. The cutoff for positive DENV-1 samples was set at a Cq detection of 35 PCR cycles [47, 48].

### Wing length

Wing length was used as an indicator of mosquito female body size [49]. Dissected wings from mosquitoes were mounted on microscope glass slides with double sided tape and a coverslip. Wing length (mm), from the base of the alula to the wing apex, was measured from photos taken using a microscope and image analysis software calibrated with a micrometer (AMScope Microscope Digital Camera).

### Nutrient stoichiometry and isotope analyses

The dissected head and thorax of *Ae. aegypti* females, preserved at −80 °C, were dried in an oven at 50 °C for 48 h. Females from larval density × detritus combinations were pooled by replicate and encapsulated (5 × 9 mm tin capsule) for nutrient stoichiometry (%C, %N, and C:N) and isotope analysis (δ^13^C and δ^15^N). Encapsulated samples weighed between 1 and 3 mg. Because there was a shortage of dissected females to reach the desired minimum sample weight of 1 mg per replicate, whole males from every replicate were encapsulated separately from females and sent for nutrient analyses. Encapsulated samples were sent to the U.S. Environmental Protection Agency, National Health and Environmental Effects Research Laboratory in Narragansett, RI, USA for nutrient analyses. Samples were analyzed for nutrients and isotopes using an ISOPRIME 100 IRMS with a Vario ISOTOPE Cube elemental analyzer inlet [19], with precision of the laboratory standards being better than ± 0.3‰ for C and N.

We acknowledge that the majority of lipid and carbohydrate reserves in *Ae. aegypti* are found in the abdomen [50, 51], and that excluding female abdomens from our nutrient analyses due to low sample size for testing both DENV-1 and nutrients on a replicate level represents a limitation. Studies have reported sex differences in *Ae. aegypti* energy reserves, with females accumulating higher lipid [52] and carbohydrate [53] levels than males. However, other studies have found no significant differences in C:N ratios [16] or δ^13^C and δ^15^N [54] between male and female *Ae. aegypti* within the same larval treatment. Consistent with this, we compared male whole-body nutrients to female head and thorax nutrients and observed no statistically significant differences in nutrients by sex. This approach allowed us to retain all replicates for both virus and nutrient analyses, preserving statistical power.

### Statistical analysis

All statistical analyses were conducted using Rstudio 2025.09.2 + 418 [55]. Statistical assumptions of normality and homogeneity of variances were assessed in the dataset. Normality of residuals was evaluated using the Shapiro–Wilk test, and homogeneity of variance across treatment groups was assessed with Levene’s test. No significant violations from these assumptions were detected. Although replication varied among treatments due to differential mortality, MANOVAs and ANOVAs were used to evaluate treatment effects, which are generally robust to moderate imbalance in sample sizes [56–58]. MANOVA was performed to determine the effect of detritus, larval density, and their interaction on female *Ae. aegypti* survival (proportion of original cohort, calculated assuming a 1:1 sex ratio) from eclosion to adult emergence, development time (days) until adult emergence, and female wing length (mm). Standardized canonical coefficients were used to determine the effect of predictor variables on the variance of significant response variables. Tukey’s honest significant difference (HSD) tests were performed to determine significant differences between the groups of significant predictor variables. Female nutrient data had a low sample size on a replicate level (*n* = 20 observations). As no significant treatment differences in *Ae. aegypti* body %N, C:N, or δ^15^N between male and females were observed (Table 3), we used male data (*n* = 37) as a proxy for female nutrient variables to compensate for the low female sample size. Due to sex-specific treatment differences in %C (ANOVA: df = 9, *F* = 2.25, *P* = 0.03), female data was used for %C analyses. MANOVA was performed to determine the effect of detritus, larval density, and their interaction on female *Ae. aegypti* %C, %N, C:N, and δ^15^N. Standardized canonical coefficients were used to determine the effect of predictor variables on the variance of significant response variables [59]. Tukey’s HSD tests were performed to determine significant differences between the groups of significant predictor variables. Because δ^13^C and δ^15^N were negatively correlated (Pearson correlation: *r* = −0.85, *t* = −4.60, df = 8, *P* = 0.001), δ^13^C was excluded from the statistical analysis.

**Table 3:** ANOVA results showing that sex × treatment (detritus × density) interactions were not significant for *Ae. aegypti* body nitrogen (%N), carbon:nitrogen (C:N), and δ^15^N

Predictor variable (s)	Response variable	df	*F*	*P*-value
Sex × treatment	N	9	1.06	0.41
Sex × treatment	CN	9	1.07	0.40
Sex × treatment	δ^15^N	9	0.24	0.98

Full regression models including all predictors and intercepts are provided in Supplementary Material 1:Tables S1-S3 to show all main effects and interactions, although the sex × treatment interaction was not significant in any model

The susceptibility to infection is the proportion of all blood-fed mosquitoes with DENV-1 RNA present in their abdomens. Disseminated infection is the proportion of mosquitoes with infected abdomens that have DENV-1 RNA present in their legs. Saliva infection (proxy for transmission) is the proportion of mosquitoes with infected legs that have DENV-1 RNA present in their saliva. Three separate logistic regressions were used to determine the effect of detritus and larval density treatment on DENV-1 (1) susceptibility to infection, (2) disseminated infection, and (3) transmission (saliva infection). DENV-1 infection, dissemination, and transmission probability rates were calculated using the following formula:

$$P=\frac{1}{1+{e}^{-intercept}}$$

where *P* represented the probability and the intercept for each treatment was obtained from the logistic regression results. A total of 279 *Ae. aegypti* females were processed in the DENV-1 infection study (Table 4). As no females survived in 30:30 high and 60:60 high treatments, they were excluded from the analyses. Infection results are interpreted only for treatments in which mosquitoes survived. Some replicates had low numbers of females that ingested the infected bloodmeal. It may have been more difficult to detect significance on a replicate level given that the proportion of infected is more variable in treatment replicates with low numbers of samples. Logistic regressions were conducted at the level of individual blood-fed females. Because the model estimates effects at the level of each observation, it is not constrained by unequal or unbalanced group sizes, allowing for robust inference despite differences in replication among treatments. Pairwise comparisons between treatment combinations were performed on estimated marginal means (emmeans) derived from the logistic regression model, using Tukey, Holm, and false discovery rate (FDR) adjustments for multiple testing. Additionally, Dunnett-adjusted comparisons were conducted using the 30:0 low treatment as the reference. Replicates were not included as a random effect due to the several replicates that contained few engorged females, resulting in unstable or nonestimable variance in mixed-effects models. We acknowledge that this approach may increase the risk of pseudoreplication, as individuals within the same container share environmental conditions beyond treatment effects, and therefore results from individual-level analyses should be interpreted with this limitation in mind. Consequently, individual mosquitoes cannot be considered fully independent observations, and treatment effects estimated at the individual level may be overstated. Therefore, inferences should be interpreted as reflecting treatment effects at the container level rather than strictly at the level of individual mosquitoes. Despite this limitation, treatment effects were consistent and biologically coherent across analyses.

**Table 4:** *Aedes aegypti* females processed by treatment in DENV-1 infection study

Treatment	# of females
30:0L	33
30:0 M	22
30:0H	20
60:0L	66
60:0 M	36
60:0H	28
30:30L	18
30:30 M	14
30:30H	0
60:60L	21
60:60 M	21
60:60H	0

Treatment was the interaction between larval density (*Ae. aegypti*:*Ae. mediovittatus*) and detritus level, represented as *L* low, *M*medium, *H* high

To examine the relationship between *Ae. aegypti* body nutrient content and DENV-1 infection, dissemination, and transmission, we used zero-inflated binomial (ZIB) regression models implemented in glmmTMB [60]. Many containers included mosquitoes with no detectable DENV-1 infection, resulting in an excess of zeros relative to standard binomial expectations. Zero-inflated binomial (ZIB) models explicitly account for these zero-inflation, improving model fit and allowing for a more accurate estimation of the effects of nutrient variables on infection outcomes, as supported by Akaike information criterion (AIC) comparisons [60, 61]. Replicates were treated as independent sampling units with each replicate contributing as one observation to the zero-inflated regression models. %N and C:N were strongly negatively correlated (Pearson correlation: *r* = −0.96, *t* = −22.18, df = 35, *P* < 0.001), thus C:N was excluded from the analyses. One model included %N and δ^15^N as predictors (*n* = 37; male nutrient values used as proxies for females), and the other used %C alone (*n* = 20; female data). Natural splines were applied to predictors to capture potential nonlinear relationships, with degrees of freedom selected via AIC.

## Results

### Detritus and larval density on female phenotypic traits

**Fig 1.**
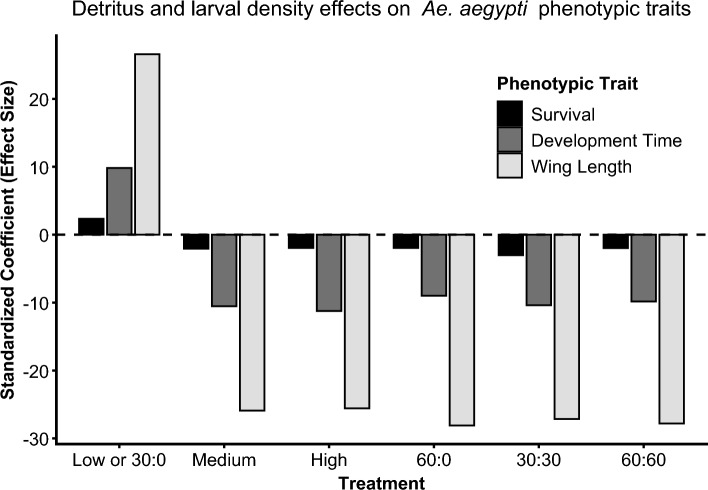
Standardized coefficients derived from MANOVA canonical structure showing the effects of detritus level and larval density on *Ae. aegypti* phenotypic traits. Values represent effect sizes relative to the baseline treatment (low detritus and 30:0 density). The dashed line at zero indicates no effect

Female *Ae. aegypti* phenotypic traits (survival, development time, and wing length) showed significant differences by detritus treatment (MANOVA: Pillai Trace_6,50_ = 0.63, *P* = 0.002) and larval density (MANOVA: Pillai Trace_9,78_ = 0.85, *P* = 0.001), but not their interaction (MANOVA: Pillai Trace_12,78_ = 0.28, *P* = 0.770). Treatment effects were strongest on development time. *Aedes aegypti* female development time was shorter in high detritus compared with low detritus treatments (Tukey HSD: M = −1.07, *P* = 0.002). *Aedes aegypti* females from interspecific competition 30:30 exhibited the shortest development time while females from high-density intraspecific competition 60:0 showed the longest (Table 5, Fig. 1). Wing length had a positive relationship with detritus treatment (Table 5), with longer wings observed in *Ae. aegypti* females from high detritus compared with low detritus (Tukey HSD: *M* = 0.11, *P* = 0.020). For wing length, all larval density treatments had negative relationships compared with low-density intraspecific competition 30:0 (Table 5, Fig. 1). Shorter wings were observed in females from high-density intraspecific competition 60:0 (Tukey HSD: *M* = −0.12, *P* = 0.004) and high-density interspecific competition 60:60 (Tukey HSD: *M* = −0.14, *P *= 0.02) compared with low-density intraspecific competition 30:0 (Fig. 1). When total larval density was the same, *Ae. aegypti* from the intraspecific competition treatment 60:0 had longer development time and shorter wings compared with the interspecific competition treatment 30:30 (Table 5). *Aedes aegypti* survival had a strong, negative relationship with interspecific competition 30:30 (Table 5), however, Tukey HSD tests showed no significant effects (*P* > 0.05) of survival by detritus or density. Although not statistically significant, no *Ae. aegypti* females from treatment combinations of 30:30 high and 60:60 high survived to adulthood.

**Table 5:** Standardized regression coefficients from MANOVA showing treatment effect sizes on female *Ae. aegypti* phenotypic traits

Treatment	Survival	Development time	Wing length
Low or 30:0 (baseline)	2.34	9.82	26.57
Medium	0.27	−7.01	0.67
High	0.39	−1.40	0.99
60:0	0.40	8.42	−1.51
30:30	−0.67	−5.61	−0.56
60:60	0.37	1.63	−1.22

Standardized regression coefficients from the MANOVA represent the direction and magnitude of treatment effect sizes, expressed in standard deviation units, relative to baseline treatments (low detritus and 30:0 larval density). Coefficients shown for baseline treatments correspond to model intercepts rather than observed outcome values. Treatments represent either detritus level (low, medium, high) or larval density (*Ae. aegypti*:*Ae. mediovittatus*)

### Detritus and larval density on nutrient profile

**Fig 2.**
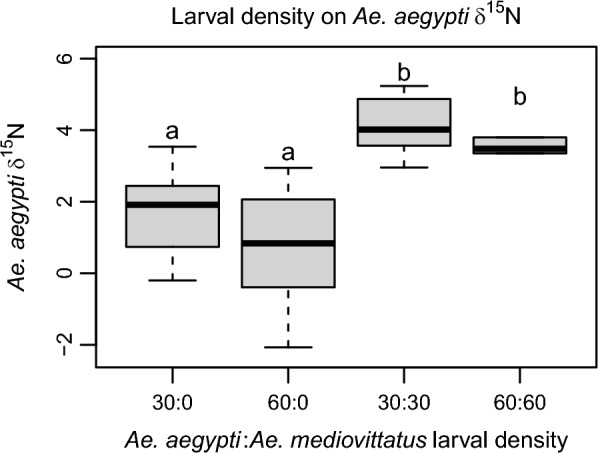
Effects of larval density on *Ae. aegypti* δ^15^N. Boxplots show δ^15^N values of *Ae. aegypti* reared under four *Ae. aegypti*:*Ae. mediovittatus* larval density treatments. The box represents the interquartile range (25th–75th percentiles), the line inside the box indicates the median, and the whiskers extend to the minimum and maximum values. Different letters above boxes represent significant differences among treatments on the basis of Tukey HSD post hoc comparisons

Female *Ae. aegypti* nutrient profile (%N, C:N) and stable isotopes (δ^15^N) showed significant differences by larval density (MANOVA: Pillai Trace_3,84_ = 1.01, *P* < 0.001), detritus treatment (MANOVA: Pillai Trace_2,54_ = 0.68, *P* < 0.001), and their interaction (MANOVA: Pillai Trace_6,84_ = 0.89, *P* = 0.01). Follow-up ANOVAs showed that the interaction of detritus and density significantly influenced N and C:N, and that separately, detritus and density treatment significantly influenced δ^15^N (Table 6). However, post hoc Tukey HSD tests only showed a significant effect of density and detritus on δ^15^N. Regardless of total larval density, *Ae. aegypti* from interspecific competition treatments (30:30 and 60:60) showed significantly higher δ^15^N than those from intraspecific competition treatments (30:0 and 60:0) (Fig. 2; Tukey HSD: *P* < 0.05). When total larval density was the same, *Ae. aegypti* from interspecific competition 30:30 had higher δ^15^N than *Ae. aegypti* from intraspecific competition 60:0. Higher δ^15^N (‰) was observed with increasing detritus, with a mean difference of 1.31‰ between medium and low detritus treatment (Tukey HSD: *P* < 0.05) and a mean difference of 1.75‰ between high and low detritus treatments (Tukey HSD: *P* < 0.05).

**Table 6:** ANOVA results of detritus and larval density effects on *Ae. aegypti* body nutrient variables

Predictor variable (s)	Response variable	DF	*F*	*P*-value
Detritus × density	N	6	4.30	0.003*
Detritus × density	CN	6	3.60	0.009*
Density	δ^15^N	3	28.02	< 0.001*
Detritus	δ^15^N	2	15.38	< 0.001*

^*^Statistically significant

### Detritus, density, and nutrients on DENV-1 vector competence

**Fig 3.**
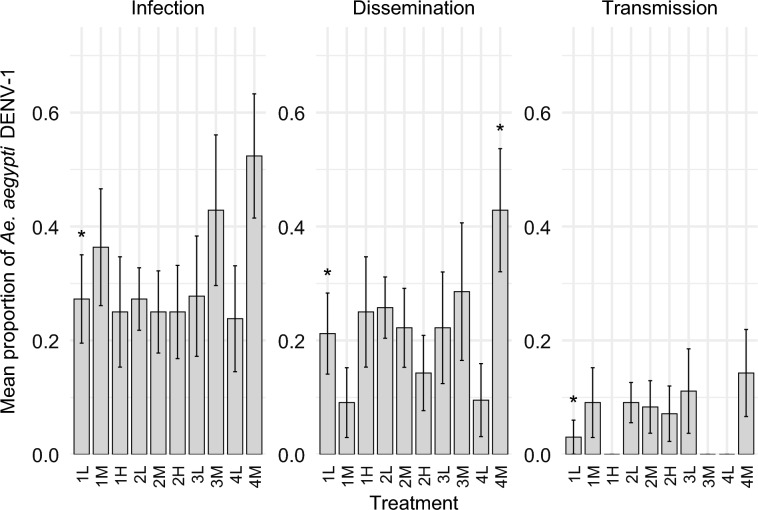
Mean proportion of *Ae. aegypti* females positive for infection, disseminated infection, and transmission of dengue virus serotype 1 across treatments. Treatments represent combinations of larval density of *Ae. aegypti* and *Ae. mediovittatus* (density: 1 = 30:0, 2 = 60:0, 3 = 30:30, 4 = 60:60) and detritus level (L = low, M = medium, H = high). The bars show mean proportions ± SE, but the underlying dataset captures outcomes for each individual mosquito, reflecting variation among mosquitoes. Asterisks (*) indicate treatments with significant differences determined by logistic regressions. Significant differences may not always be obvious and logistic regression analyzes data on a logit (nonlinear) scale, whereas the bars show raw proportions. Infection status was determined for each individual mosquito using a qRT-PCR Cq threshold (≤ 35). This approach detects viral RNA in the sample

**Fig 4.**
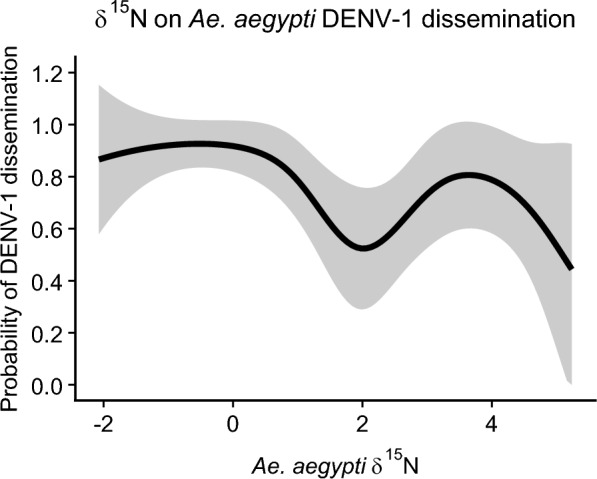
Zero-inflated binomial regression showing the relationship between *Ae. aegypti* δ^15^N and DENV-1 dissemination. The solid line represents the predicted probability of dissemination across the observed δ^15^N range, and the shaded area indicates the 95% confidence interval around the predictions

*Aedes aegypti* females from 30:0 low showed lower susceptibility to DENV-1 infection (logistic regression: estimate = −0.98, *P* = 0.01), lower disseminated infection (logistic regression: estimate = −1.31, *P* = 0.002), and lower transmission (logistic regression: estimate −3.46, *P* < 0.001) compared with females from other treatments. The predicted probabilities of DENV-1 infection, dissemination, and transmission in *Ae. aegypti* from 30:0 low were 27.27%, 21.22%, and 3.03%, respectively. Females from 60:60 medium exhibited higher rates of disseminated infection of DENV-1 (estimate = 2.95, *P* = 0.01) than females from other treatments (Fig. 3). Pairwise comparisons across all treatment combinations using Tukey, Holm, or FDR adjustments did not detect additional significant differences, likely due to the small sample size and the conservative nature of multiple testing corrections. The predicted probability of DENV-1 dissemination for females from 60:60 medium was 95.04%. Zero-inflated binomial regression models only showed a significant relationship between *Ae. aegypti* body δ^15^N and DENV-1 dissemination (estimate = −2.90, *Z* = −2.25, *P* = 0.02). DENV-1 dissemination exhibited a nonlinear relationship with δ^15^N, with high dissemination at low δ^15^N values, the lowest dissemination at intermediate δ^15^N, and a partial increase of dissemination at higher δ^15^N (Fig. 4).

## Discussion

This study examined how detritus availability and larval competition affects *Ae. aegypti* phenotypic traits (wing length), nutrient composition, and vector competence for DENV-1. We hypothesized that low detrital inputs and increased interspecific competition with *Ae. mediovittatus* would negatively influence phenotypic traits and decrease body carbon and nitrogen content. We further predicted that these nutrient limitations would increase VC for DENV-1, linking larval environmental stressors to adult vector competence. As we hypothesized, low detritus environments decreased female *Ae. aegypti* wing length, increased development time, had little influence on survival, and were associated with less *Ae. aegypti* body δ^15^N. A positive relationship between total detritus amount and female *Ae. aegypti* wing length was observed, a trait that is representative of female body size. Less detritus in containers has been associated with smaller (lower biomass) field collected [18] and laboratory-reared adult *Ae. aegypti* [62, 63]. Similarly, a positive relationship between *Ae. aegypti* biomass and invertebrate carcass detritus has been previously shown [63]. Here we show that treatments with increasing total detritus—co-occurring with greater inputs of animal detritus and increased detritus diversity—were associated with larger female *Ae. aegypti* wing lengths and elevated body δ^15^N, suggesting combined effects of resource quantity and quality. We highlight how nutrients in the body tissues of *Ae. aegypti* mirror nutrients that enter the larval environments from detritus sources with effects on the species phenotypic traits. Nitrogen content of field-collected adult females of *Ae. aegypti* has been previously related to nitrogen content in container water and leaf detritus at the sampling location [19]. Our results also support previous findings of reduced detritus inputs and associated differences in detritus composition in the larval habitat being associated with longer development time of *Ae. aegypti* [63–66]. Low resource availability for mosquito larvae can lengthen the development time necessary to reach their pupation threshold [12]. *Aedes aegypti* larvae inhabit containers that receive pulses of detrital inputs, and low resource availability can induce starvation conditions [64]. The ability of *Ae. aegypti* to withstand starvation, especially among later instars, could have caused the weak influence of detritus on female survival in this experiment, especially when compared with development time and wing length effects. *Aedes* mosquitoes have displayed similar survival across detritus types in previous studies [16].

Contrary to our hypotheses, interspecific competition with *Ae. mediovittatus* did not increase development time or reduce survival, wing length, or nitrogen content in *Ae. aegypti*. Surprisingly, *Ae. aegypti* from interspecific density (30:30) had shorter development times and longer wings than *Ae. aegypti* females from intraspecific density (60:0). These observations portray *Ae. aegypti* as a superior larval competitor to *Ae. mediovittatus* under the studied detritus level and larval density conditions. This is further supported by *Ae. aegypti* from interspecific density treatments (30:30 and 60:60) having higher δ^15^N in body tissues than intraspecific density treatments (30:0 and 60:0). Recent larval competition experiments between *Ae. aegypti* and *Ae. mediovittatus* [15] show *Ae. mediovittatus* outcompeting *Ae. aegypti* across all treatment combinations and decreasing *Ae. aegypti* survival and population growth (λ’), a measure that incorporates biomass.

The contrasting competitive outcomes of our study are likely driven by differences in detritus, including variation in detritus type, quality, and composition. Unlike earlier studies that used predominantly plant detritus [e.g., 15], including lignin-rich leaf species such as *Bambusa* spp. [67], *Cecropia schreberiana* [68], *Ficus benjamina*, and *Ficus elastica* [69], our design incorporated animal detritus across all treatments and systematically increased both total detritus and resource diversity. Animal detritus decomposes rapidly, increases microbial productivity, and provides a high nitrogen, high-quality resource base for mosquito larvae [30, 70]. In contrast, leaf litter with higher lignin content decomposes slowly and suppresses bacterial growth [71], which offers a poorer source of nutrition to mosquito larvae. Together, these differences suggest that competitive hierarchies between *Ae. aegypti* and *Ae. mediovittatus* are context dependent, varying with detritus type, quality, and microbial dynamics. Similar observations have been reported among other container-inhabiting mosquitoes where interspecific competition among larvae has been assessed [72–74]

The nonlinear relationship between *Ae. aegypti* δ^15^N and DENV-1 dissemination revealed that the lowest δ^15^N values were associated with the highest levels of viral dissemination (Fig. 4), consistent with the premise that nutritionally poor larval environments reduce immune capacity and allow for greater viral replication and systemic spread [4, 10, 21]. Because δ^15^N values for females were inferred from male proxies in some cases, and replicate-level sample sizes were modest (*n* = 37 for δ^15^N), this nonlinear relationship should be interpreted as suggestive rather than definitive. As expected, treatments with lower total detritus and reduced detritus diversity produced smaller *Ae. aegypti* females with reduced wing length, and these individuals exhibited the highest dissemination rates. However, this effect was not observed for DENV-1 infection or transmission, suggesting that nutritional stress primarily compromises postinfection immune barriers (e.g., midgut escape barrier) rather than initial midgut infection or salivary gland transmission barriers. Nutritionally constrained mosquitoes may remain susceptible to infection but lack the physiological capacity to effectively contain viral replication beyond the midgut, resulting in elevated dissemination [10] without corresponding increases in infection or transmission. Together, these findings highlight the nonlinear nature of nutritional effects on vector competence and suggest that larval nutritional stress disproportionately influences viral dissemination rather than all stages of dengue infection dynamics. These results are consistent with previous studies of *Ae. aegypti* exposed to nutrient-deprived (starved) conditions as larvae followed by adult oral ingestion of Zika virus (ZIKV)-infected blood or intrathoracic inoculation of ZIKV [20]. Transmission electron microscopy of the midgut basal lamina showed that starved mosquitoes had thinner basal lamina than optimally reared control mosquitoes. The researchers suggested that nutrient-associated impairment of the midgut escape barrier contributed to enhanced disseminated infection of ZIKV. Interestingly, intrathoracic inoculation of *Ae. aegypti*, which bypasses the midgut barriers, showed lower ZIKV titers in the head and carcasses in starved mosquitoes compared with optimally fed control mosquitoes [20]. These observations suggest that ZIKV replication is more efficient in secondary tissues of well-nourished mosquitoes than starved mosquitoes. Therefore, the way larval nutrition contributes to altered vector competence for arboviruses can differ among the components of vector competence. Along the same lines, a study manipulated the larval nutrition (low and normal) and adult nutrition (low and normal sucrose) in *Ae. aegypti* [21]. They found that both larval and adult nutritional stress suppressed immune-related genes related to Toll, Jak-STAT, and Imd immune signaling pathways, and reduced expression of antimicrobial peptides [21]. Both larval and adult nutritional stress enhanced susceptibility to infection and disseminated infection of DENV-4, suggesting that nutrition directly impacts antiviral immunity in *Ae. aegypti* and alters aspects of vector competence for dengue virus [21]. Because replicate-level variation could not be explicitly modeled due to low numbers of engorged females in some containers, individual-level analyses may include some degree of pseudoreplication; therefore, these results should be interpreted with appropriate caution.

Our lowest detritus and density treatment (30:0 low) produced the lowest DENV-1 infection, dissemination, and transmission, suggesting that in this treatment, nitrogen was not limited enough to increase *Ae. aegypti* DENV-1 dissemination. It may be that this detritus and density combination allowed for a balanced stoichiometry that enhanced immune homeostasis and reduced VC for DENV-1 via pathogen suppression in *Ae. aegypti* [21]. Although *Ae. aegypti* exhibited competitive dominance under interspecific conditions with *Ae. mediovittatus*, as evidenced by faster development and larger wing size relative to intraspecific treatments, elevated δ^15^N values suggest that this dominance may be achieved at a physiological cost. Increased δ^15^N under interspecific competition with *Ae. mediovittatus* likely reflects altered nitrogen metabolism or increased nitrogen turnover [75], potentially arising from competition in detrital environments more characteristic of the natural habitat of *Ae. mediovittatus* [18, 28, 33]. Such metabolic adjustments could impose stress on immune homeostasis in *Ae. aegypti*, reduce the effectiveness of antiviral barriers, facilitate viral escape from the midgut, and promote systemic dissemination [4, 10, 20, 21], providing a plausible mechanism linking competitive interactions, nutrient dynamics, and nonlinear patterns of DENV-1 dissemination. This interpretation is consistent with the association we observed between the highest δ^15^N values and intermediate DENV-1 dissemination in *Ae. aegypti* (Fig. 4). Additional support comes from the absence of *Ae. aegypti* survival under the combined interspecific competition (30:30 and 60:60) and high detritus, suggesting that competitive and nutritional stressors can interact to constrain fitness. Because no individuals survived under these conditions, they were necessarily excluded from infection analyses; therefore, our conclusions regarding DENV-1 infection, dissemination, and transmission apply only to treatments in which mosquitoes survived to adulthood.

We acknowledge that infection status was determined using qRT-PCR with a Cq threshold (≤ 35), and that interpretations of vector competence are based on the proportion of mosquitoes testing positive for infection rather than on direct measurement of infectious particles. As such, while high detritus combined with interspecific competition clearly imposes strong survival constraints, its effects on vector competence cannot be directly assessed in the absence of surviving individuals. Adult longevity was not measured in this study and therefore could not be directly evaluated as a component of transmission dynamics. While larval conditions may influence adult survival via carry-over effects, this limitation should be considered when interpreting overall vector competence outcomes. An additional limitation of this study is the lack of precise information regarding the generational history of the mosquito colonies. Although the source populations are periodically refreshed with field material, the undocumented generational turnover prevents a clear assessment of the extent of laboratory adaptation. This is important because laboratory colonization and adaptation can influence life-history traits, immune function, and vector competence. Nevertheless, periodic additions of wild genotypes every 3–6 months likely maintains greater genetic variability than long-established laboratory strains, supporting the biological relevance of the observed responses.

## Conclusions

Taken together, our results suggest that δ^15^N integrates underlying nutritional and competitive dynamics experienced during *Ae. aegypti* larval development and can serve as a useful marker of larval environmental conditions that shape dengue vector competence. However, we note that female-specific nutrient measurements were limited, and male whole-body values were used as proxies for female head and thorax nutrient measurements. Therefore, interpretations linking δ^15^N to female vector competence should be made with caution, and further studies directly measuring female nutrient status are needed to validate these patterns. Our findings may help explain the distribution of *Ae. aegypti* in Puerto Rico, where the presence of *Ae. mediovittatus* in rural, suburban, and forested habitats [28, 33], together with naturally high detrital inputs [18], may limit *Ae. aegypti* expansion, as previously suggested in another study [15]. These larval competitive outcomes may also influence naturally occurring vector competence dynamics between co-occurring species, as evidenced by higher DENV-1 dissemination in *Ae. aegypti* from the 60:60 medium detritus treatment in our laboratory experiments.

## Supplementary Information

Supplementary Material 1.

## Data Availability

The datasets generated and/or analyzed during the current study are available in the Dryad repository, [10.5061/dryad.280gb5n2z].

## Abbreviations

*Ae*: Aedes
*C*: Carbon
*%C*: Percent carbon
*C:N*: Carbon:nitrogen ratio
*DI*: Deionized
*DENV*: Dengue virus
*DENV-1*: Dengue virus serotype 1
*N*: Nitrogen
*%N*: Percent nitrogen
*UF-FMEL*: Florida Medical Entomology Laboratory, University of Florida
*VC*: Vector competence
