# Supplementary material for: Detritus and larval competition influence phenotypic traits, nutrient stoichiometry, and vector competence for dengue virus in Aedes aegypti
Source: Parasit Vectors. 2026 Jun 2;19:315. doi: 10.1186/s13071-026-07467-1 (PMC13440121; doi:10.1186/s13071-026-07467-1)
Supplement: Supplementary file 1 [file 13071_2026_7467_MOESM1_ESM.docx]

Model: Nitrogen ~ Sex × Treatment

| **Interaction** | **Term** | **Estimate** | **SE** | **t value** | **p-value** |
| --- | --- | --- | --- | --- | --- |
| Baseline (Males:30:0L) | (Intercept) | 6.175 | 0.381 | 16.20 | <0.001* |
| Sex | Females | 0.744 | 0.809 | 0.92 | 0.363 |
| Treatment (Density*Detritus) | 60:0Low | -0.468 | 0.591 | -0.79 | 0.433 |
|  | 30:30Low | -1.061 | 0.632 | -1.68 | 0.101 |
|  | 60:60Low | -2.085 | 0.809 | -2.58 | 0.014* |
|  | 30:0Medium | -0.823 | 0.632 | -1.30 | 0.201 |
|  | 60:0Medium | -1.769 | 0.696 | -2.54 | 0.015* |
|  | 30:30Medium | 0.137 | 0.632 | 0.22 | 0.830 |
|  | 60:60Medium | 0.461 | 0.809 | 0.57 | 0.572 |
|  | 30:0High | -1.398 | 0.591 | -2.37 | 0.023* |
|  | 60:0High | -0.490 | 0.696 | -0.70 | 0.485 |
| Sex* Treatment (Density*Detritus) | Females:60:0Low | -0.007 | 1.169 | -0.01 | 0.995 |
|  | Females:30:30Low | 1.193 | 1.190 | 1.00 | 0.322 |
|  | Females:60:60Low | 2.432 | 1.293 | 1.88 | 0.067 |
|  | Females:30:0Medium | 1.191 | 1.190 | 1.00 | 0.323 |
|  | Females:60:0Medium | 1.875 | 1.225 | 1.53 | 0.134 |
|  | Females:30:30Medium | 0.004 | 1.190 | 0.00 | 0.998 |
|  | Females:60:60Medium | 1.821 | 1.293 | 1.41 | 0.167 |
|  | Females:30:0High | 1.960 | 1.169 | 1.68 | 0.102 |
|  | Females:60:0High | 1.667 | 1.225 | 1.36 | 0.181 |

**Table S1. Full linear regression model for Nitrogen (N).** Model: N ~ Sex × Treatment. Residual standard error: 1.009 on 39 DF; R² = 0.660, Adjusted R² = 0.495. The intercept corresponds to the baseline group (Males, 30:0Low). The model coefficients (Estimate) represent differences relative to the baseline group (Males, 30:0Low). Main effect terms show differences from this baseline, and interaction terms indicate whether the effect of treatment differs between sexes. The significance of the intercept indicates that the predicted value for this baseline is different from zero and does not reflect differences from other treatments or sexes.

Model: Carbon:Nitrogen ~ Sex × Treatment

|  | **Interaction** |  | **Term** | **Estimate** | **SE** | **t value** | **p-value** |
| --- | --- | --- | --- | --- | --- | --- | --- |
|  | Baseline (Males:30:0L) |  | (Intercept) | 8.588 | 0.542 | 15.83 | <0.001* |
|  | Sex |  | Females | -1.007 | 1.151 | -0.88 | 0.387 |
|  | Treatment (Density*Detritus) |  | 60:0Low | 1.291 | 0.840 | 1.54 | 0.133 |
|  |  |  | 30:30Low | 1.550 | 0.900 | 1.72 | 0.093 |
|  |  |  | 60:60Low | 3.561 | 1.151 | 3.10 | 0.004* |
|  |  |  | 30:0Medium | 1.160 | 0.900 | 1.29 | 0.205 |
|  |  |  | 60:0Medium | 3.076 | 0.990 | 3.11 | 0.004* |
|  |  |  | 30:30Medium | -0.127 | 0.900 | -0.14 | 0.889 |
|  |  |  | 60:60Medium | -0.290 | 1.151 | -0.25 | 0.774 |
|  |  |  | 30:0High | 2.175 | 0.840 | 2.59 | 0.013* |
|  |  |  | 60:0High | 0.826 | 0.990 | 0.83 | 0.409 |
|  | Sex* Treatment (Density*Detritus) |  | Females:60:0Low | -0.547 | 1.663 | -0.33 | 0.744 |
|  |  |  | Females:30:30Low | -1.734 | 1.694 | -1.02 | 0.312 |
|  |  |  | Females:60:60Low | -2.926 | 1.839 | -1.59 | 0.120 |
|  |  |  | Females:30:0Medium | -1.104 | 1.694 | -0.65 | 0.518 |
|  |  |  | Females:60:0Medium | -3.009 | 1.744 | -1.73 | 0.092 |
|  |  |  | Females:30:30Medium | 0.572 | 1.694 | 0.34 | 0.737 |
|  |  |  | Females:60:60Medium | -0.531 | 1.839 | -0.29 | 0.774 |
|  |  |  | Females:30:0High | -3.093 | 1.663 | -1.86 | 0.070 |
|  |  |  | Females:60:0High | -1.670 | 1.744 | -0.96 | 0.344 |

**Table S2. Full linear regression model for Carbon:Nitrogen (CN).** Model: CN ~ Sex × Treatment**.** Residual standard error: 1.435 on 39 DF; R² = 0.606, Adjusted R² = 0.414. The intercept corresponds to the baseline group (Males, 30:0Low). The model coefficients (Estimate) represent differences relative to the baseline group (Males, 30:0Low). Main effect terms show differences from this baseline, and interaction terms indicate whether the effect of treatment differs between sexes. The significance of the intercept indicates that the predicted value for this baseline is different from zero and does not reflect differences from other treatments or sexes.

Model: δ¹⁵N ~ Sex × Treatment**.**

| **Interaction** | **Term** | **Estimate** | **SE** | **t value** | **p-value** |
| --- | --- | --- | --- | --- | --- |
| Baseline (Males:30:0L) | (Intercept) | 0.862 | 0.299 | 2.888 | 0.006* |
| Sex | Females | 0.038 | 0.634 | 0.059 | 0.953 |
| Treatment (Density*Detritus) | 60:0Low | -1.363 | 0.463 | -2.946 | 0.005* |
|  | 30:30Low | 3.032 | 0.495 | 6.121 | <0.001* |
|  | 60:60Low | 1.275 | 0.634 | 2.012 | 0.051 |
|  | 30:0Medium | 0.980 | 0.495 | 1.979 | 0.055 |
|  | 60:0Medium | 0.386 | 0.545 | 0.708 | 0.483 |
|  | 30:30Medium | 3.743 | 0.495 | 7.557 | <0.001* |
|  | 60:60Medium | 3.156 | 0.634 | 4.982 | <0.001* |
|  | 30:0High | 1.746 | 0.463 | 3.774 | <0.001* |
|  | 60:0High | 1.440 | 0.545 | 2.641 | 0.012* |
| Sex* Treatment (Density*Detritus) | Females:60:0Low | -0.067 | 0.916 | -0.073 | 0.942 |
|  | Females:30:30Low | -0.357 | 0.933 | -0.382 | 0.704 |
|  | Females:60:60Low | 0.827 | 1.013 | 0.816 | 0.419 |
|  | Females:30:0Medium | -0.165 | 0.933 | -0.177 | 0.860 |
|  | Females:60:0Medium | 0.151 | 0.960 | 0.157 | 0.876 |
|  | Females:30:30Medium | -0.178 | 0.933 | -0.191 | 0.849 |
|  | Females:60:60Medium | -0.020 | 1.013 | -0.020 | 0.984 |
|  | Females:30:0High | 0.125 | 0.916 | 0.136 | 0.892 |
|  | Females:60:0High | 0.628 | 0.960 | 0.654 | 0.517 |

**Table S3. Full linear regression model for δ¹⁵N.** Model: δ¹⁵N ~ Sex × Treatment**.** Residual standard error: 0.790 on 39 DF; R² = 0.850, Adjusted R² = 0.777. The intercept corresponds to the baseline group (Males, 30:0Low). The model coefficients (Estimate) represent differences relative to the baseline group (Males, 30:0Low). Main effect terms show differences from this baseline, and interaction terms indicate whether the effect of treatment differs between sexes. The significance of the intercept indicates that the predicted value for this baseline is different from zero and does not reflect differences from other treatments or sexes.
